# Case Report: Necrotizing Stomatitis as a Manifestation of COVID-19-Associated Vasculopathy

**DOI:** 10.3389/fped.2021.800576

**Published:** 2021-12-13

**Authors:** Nina Emeršič, Tanja Tomaževič, Olga Točkova, Matjaž Kopač, Metka Volavšek, Damjana Ključevšek, Tadej Avčin

**Affiliations:** ^1^Department of Allergology, Rheumatology and Clinical Immunology, Children's Hospital, University Medical Center Ljubljana, Ljubljana, Slovenia; ^2^Department of Pediatric and Preventive Dentistry, Dental Clinic, University Medical Center Ljubljana, Ljubljana, Slovenia; ^3^Department of Dermatovenereology, University Medical Center Ljubljana, Ljubljana, Slovenia; ^4^Department of Nephrology, Children's Hospital, University Medical Center Ljubljana, Ljubljana, Slovenia; ^5^Faculty of Medicine, Institute of Pathology, University of Ljubljana, Ljubljana, Slovenia; ^6^Radiology Unit, Children's Hospital, University Medical Center Ljubljana, Ljubljana, Slovenia; ^7^Faculty of Medicine, University of Ljubljana, Ljubljana, Slovenia

**Keywords:** necrotizing stomatitis, vasculitis, hypertension, SARS-CoV-2, COVID-19 associated vasculopathy, case report, damage of endothelial cell

## Abstract

Necrotizing stomatitis is a rare, acute-onset disease that is usually associated with severely malnourished children or diminished systemic resistance. We describe a 1-year-old girl who developed necrotizing stomatitis, vasculitic rash, skin desquamation on the fingers and toes, and persistent hypertension after serologically confirmed SARS-CoV-2 infection. Her laboratory investigations revealed positive IgG anticardiolipin and IgG anti-β2 glycoprotein antibodies, and biopsy of the mucosa of the lower jaw showed necrosis and endothelial damage with mural thrombi. Swollen endothelial cells of small veins in the upper dermis were confirmed also by electron microscopy. As illustrated by our case, necrotizing stomatitis may develop as a rare complication associated with SARS-CoV-2 infection and can be considered as a part of the clinical spectrum of COVID-19 vasculopathy. The pathogenic mechanism could involve a consequence of inflammatory events with vasculopathy, hypercoagulability, and damage of endothelial cells as a response to SARS-CoV-2 infection.

## Introduction

SARS-CoV-2 infection is asymptomatic in 15–42% of infected children (1). In symptomatic children, the main clinical manifestations are fever, cough, headache, nausea/vomiting, diarrhea, shortness of breath, sore throat, myalgia, rhinorrhea, abdominal pain, and loss of smell and taste (2, 3).

Since the outbreak of COVID-19, there is a growing number of reports on various skin and mucosal changes associated with COVID-19 as well. The pathogenesis of COVID-19 vasculopathy is most likely thrombotic lymphocytic vasculitis, and the disease has in most cases a benign course (4).

Necrotizing stomatitis (NS) is a rare, acute-onset disease with painful, destructive necrosis and ulceration in gingival, periodontal, and other oral surfaces beyond mucogingival junction. It rarely occurs in pediatric patients and is usually associated with severely malnourished children or diminished systemic resistance such as with human immunodeficiency virus (HIV) infection (5).

We report a case of a 1-year old girl with NS that developed after SARS-CoV-2 infection. To our knowledge, NS and bone necrosis in a child in association with COVID-19 has not yet been described.

## Case Description

We present a case of a 1-year-old Caucasian girl, whose past medical history was unremarkable. Two and a half months before the initial presentation, the first tooth (lower incisor) erupted and soon after 11 more teeth erupted in the upper and lower jaw.

Two months before admission, the girl traveled abroad with her family. The family negated symptoms of SARS-CoV-2 infection.

The girl initially developed an itchy skin rash. Two weeks later, she developed diarrhea which lasted for 6 days. A pediatrician also noticed acute otitis media and signs of acute stomatitis. Laboratory results showed elevated C-reactive protein (CRP, 60 mg/l) and leukocytosis.

One month after the initial presentation and one week after the resolution of diarrhea, her medical condition worsened, she became irritable, refused to eat solid food, and suddenly lost three deciduous incisors from the lower jaw. The rest of her teeth in the lower jaw were mobile. There was no report of injury or elevated temperature.

Family history was unremarkable.

### Clinical Findings

**Figure 1:**
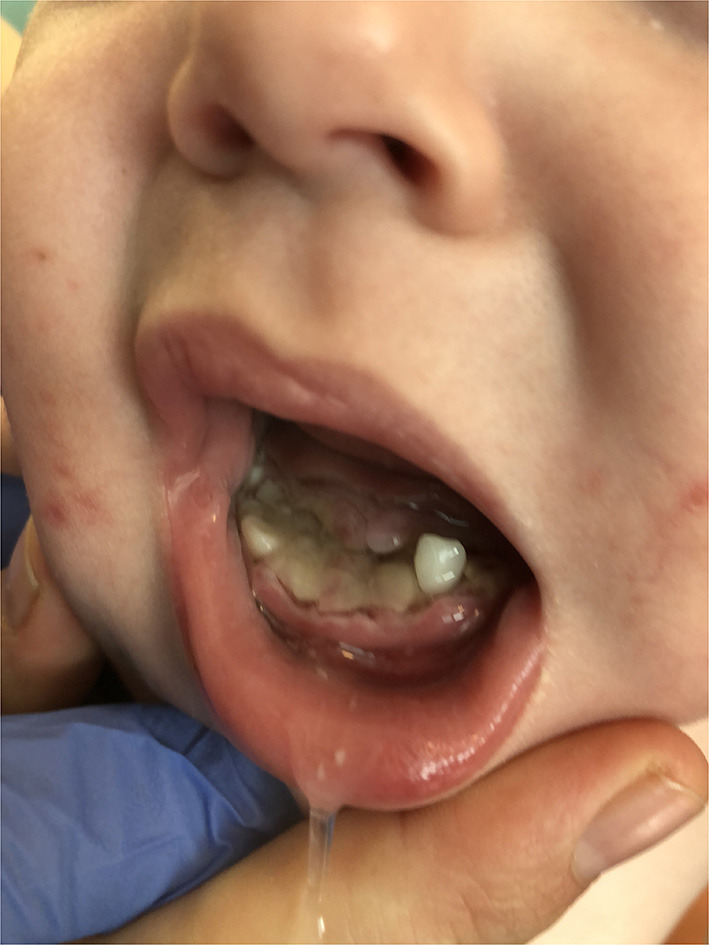
Necrosis of the frontal region of the mandibular alveolar ridge and three missing incisors.

At admission to our hospital, she was hemodynamically stable, afebrile, and irritable. Her heart rate was 176 beats/min. She presented with extensive necrosis of the frontal region of the mandibular alveolar ridge extending to deciduous molars and was missing three incisors (Figure 1). The affected area was swollen and exposed, with extensive fibrin coating. Maxillary gingiva showed no signs of gingival or periodontal inflammation. There were some aphthous changes on the tongue and buccally. On the skin, there was a generalized erythematous maculopapular rash with petechiae and scratches on the trunk and buttock. Desquamation of the skin on the fingers and edema of the wrists and ankles was also seen. There was redness of the perigenital region. Peripheral lymph nodes were not enlarged. Neurologic examination was normal.

During hospitalization, the second left incisor in the mandible fell out, the girl had cold limbs, and occasionally a reticular pattern was seen on the skin. Transiently, a wound between the second and third toes on the left foot was present.

The girl had just one peak temperature of 38.2°C; otherwise, she was afebrile and cardiovascularly compensated. On the third day after admission, she developed hypertension with systolic blood pressure between 125 and 140 mmHg and diastolic blood pressure between 60 and 90 mmHg (above the 95th percentile for age and height, which is 105/60 mmHg). She also had a persistent tachycardia with a pulse rate between 150 and 180 beats/min.

### Diagnostic Assessment

#### Laboratory Tests and Serology

In blood laboratory test results, there was a mild elevation of CRP (20 mg/l), negative procalcitonin, leukocytosis, and thrombocytosis. L*actate dehydrogenase* (LDH), liver transaminases, and fibrinogen were elevated as well. Thyroid hormones (T3 and T4) were slightly elevated.

Nasopharyngeal swab was positive for rhinovirus and enterovirus infections but was negative for other viruses including SARS-CoV-2. The swabs of the oral cavity and mandible were negative for pathogenic bacteria and herpesviruses. Skin swabs from several places showed S. aureus and Candida albicans superinfection and a vaginal swab showed the presence of Candida albicans.

The serology for SARS-CoV-2 was found positive for both IgG and IgA antibodies (IgG: 1.88; IgA: 2.17). Blood culture was negative. Her immune status was unremarkable, and primary immunodeficiencies were ruled out with laboratory tests. The girl had normal lymphocyte subpopulations, at presentation minimally elevated IgG levels, and normal IgM and IgA, normal test of phagocytosis, and normal values of complement. Immunoserology testing revealed positive IgG anticardiolipin antibodies (aCL) (12 AUG) and IgG anti-beta2-glycoprotein I antibodies (anti-ß2GPI) [16 AUG]). Vitamin A deficiency, herpes simplex virus 1 and 2, varicella zoster virus, HIV, rubella, and measles infections were ruled out. The urine sample was unremarkable. The plasma renin level was normal for the girl's age [7.3 μg/l/h (ref.: 0–3 years: <16.6 μg/l/h)]. The heart breakdown enzymes were normal.

#### Imaging

**Figure 2:**
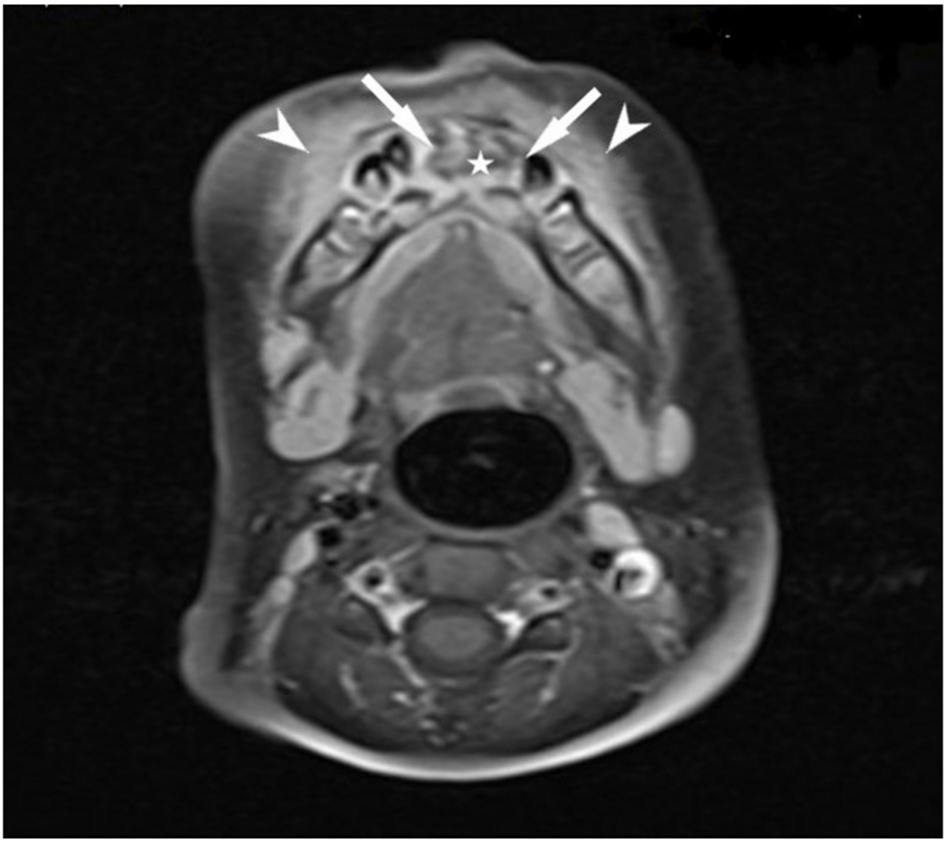
Magnetic resonance imaging of the girl's mandible (T1 TSE fat sat contrast medium sequence) showed areas of bone necrosis (star) with peripheral reactive bone contrast enhancement (arrows) and enhancement of the soft tissue edema (arrowheads).

CT and MR imaging of the lower jaw showed diffusely altered bone with edema and necrosis of the mandibular corpus and the above alveolar ridge in the region extending from tooth 84 to tooth 74 (Figure 2). It showed severe inflammation of the surrounding soft tissues and muscles as well. There was loss of teeth 71, 81, and 82.

Ultrasound of the abdomen showed slightly enlarged liver. Doppler ultrasound of renal arteries was normal, and thyroid ultrasound was normal as well. Chest X-ray was normal. Electrocardiogram and echocardiogram showed no abnormalities.

#### Histopathological Evaluation

**Figure 3:**
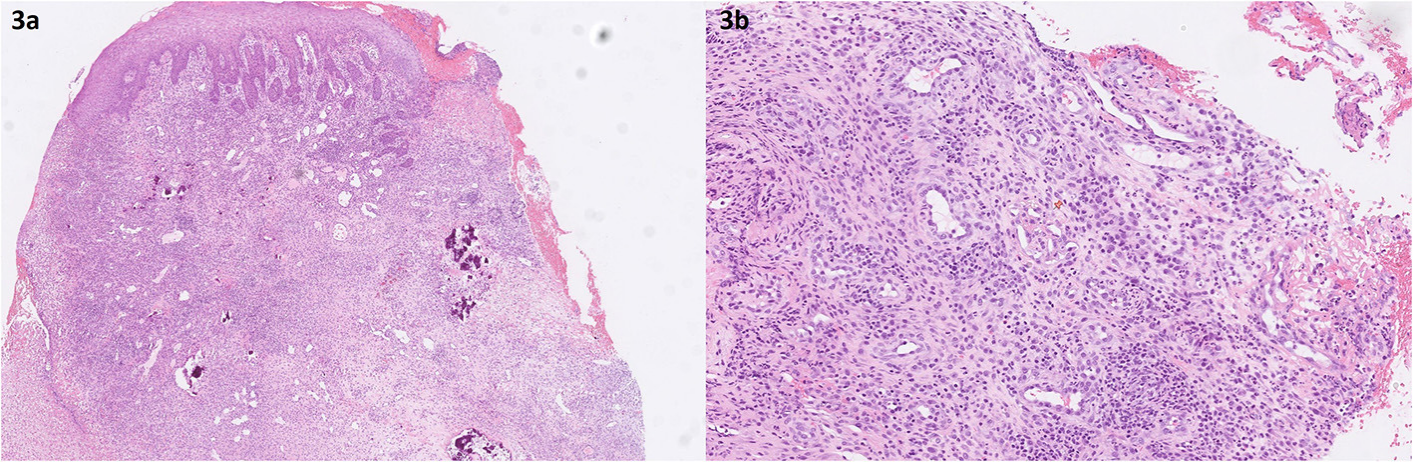
Histopathology of the biopsy specimens taken from the lower jaw (first biopsy). **(a)** Inflamed gingival mucosa with superficial erosions, with granulation tissue and dystrophic calcifications. Retrospectively, calcifications were “reinterpreted” as remains of destructed alveolar bone. HE, original magnification, ×4. **(b)** Recanalization of the small vessels in the inflamed gingival mucosa (mark). Note the intense mixed inflammatory infiltrate in the background and the swollen endothelial cells. HE, original magnification, ×20.

Biopsy of the mucosa of the lower jaw, tooth, mandible (twice), and skin was performed. During biopsy, teeth 83 and 73 were removed due to total loss of supporting alveolar bone. Histopathologically (Figure 3a), biopsy of the mucosa of the lower jaw showed necrosis, granulation tissue, and changes compatible with epulis, corresponding partly to pyogenic granuloma and partly to peripheral ossifying fibroma. No diagnostic changes for lymphoma or Langerhans cell histiocytosis were present. Immunohistochemistry for SARS-CoV-2 was negative. Necrosis and endothelial damage with individual mural thrombi and in some cases the appearance of recanalization of thrombotic changes along small vessels were seen (Figure 3b).

Biopsy of the mandible showed vital reactive bone with fibrosis without suppurative inflammation indicative of active infection.

Biopsy of the tooth showed normal structures of the tooth with adjacent structures of the alveolar ridge.

The second biopsy of the soft tissue of the mandible showed minimally reactive bone, and reactive spindle-cell proliferation with individual multinucleated giants of the osteoclast type. Compared with the previous biopsy, the changes were more chronic in appearance with reparative inflammation, without granulation tissue or necrosis.

**Figure 4:**
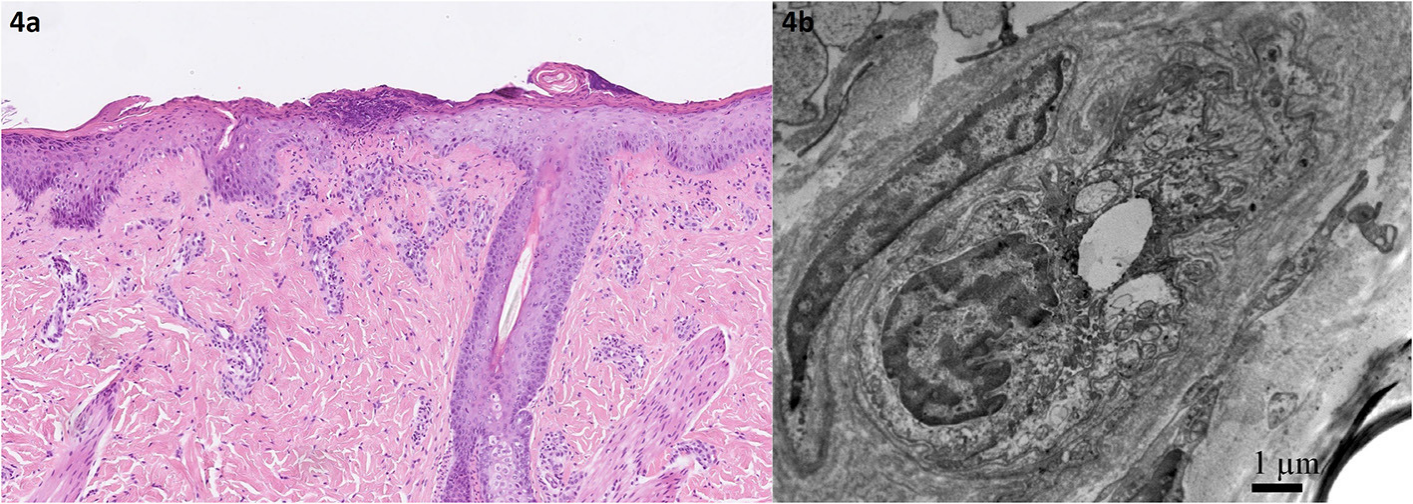
Histopathology of the skin efflorescence in the sacral area. **(a)** Focal epidermal necrosis is present in the center. Note the pronounced underlying small vessels with swollen endothelial cells in the papillary dermis. HE, original magnification, ×6. **(b)** Electron microscopy of swollen endothelial cells obliterating the lumen of the aforementioned small veins.

The samples of skin changes in the sacral area were taken and sent for analysis and possible bacterial and fungal infections. Histopathologically, sacral focal necrosis of the epidermis and swelling of endothelial cells of the dermis were seen (Figure 4a). There was no thrombosis and no visible changes typical for malignancy, histiocytosis, or active infection. Stevens–Johnson syndrome (SJS) was also excluded based on the skin-biopsy result. Swollen endothelial cells of small veins in the upper dermis that almost or completely close the lumen were confirmed by electronic microscopy (Figure 4b). There were no fibrins or platelets in the lumen of the examined vessels and on the endothelium. Endothelial cell organelles were unremarkable. No viral particles were detected. Immunofluorescence examination was unspecific.

#### Genetic Testing

The results of molecular genetic testing by the next-generation sequencing (NGS) method showed no changes in the 4,813 analyzed genes, including 304 genes associated with primary immunodeficiencies, genes associated with COVID-19 vasculitis (*TREX1*), type I interferonopathies (*STING1, COPA*), and monogenic vasculitis (*DADA2*) (6, 7).

### Treatment

The patient was treated with combined antibiotic therapy with amoxicillin and clavulanic acid intravenously. On the 13th day of hospitalization, we also introduced metronidazole 100 mg q 8 h orally and clindamycin 100 mg q 8 h intravenously and later orally. Because of the suspicion for hypercoagulability, she received antiplatelet therapy with 50 mg of acetylsalicylic acid daily. For the skin, she received ointment with a combination of corticosteroid, antibiotic, and antifungal active substance due to proven superinfection with S. aureus and Candida albicans. Against itching, she received loratadine twice daily. Against oral pain, we applied 1% lidocaine chloride locally three times a day. She needed analgesia (paracetamol 100 mg/6 h) and, due to food refusal, parenteral hydration. Due to tachycardia and arterial hypertension, she received 1 mg of antihypertensive drug amlodipine (0.1 mg/kg) twice daily.

### Follow-Up and Outcomes

Two weeks after the beginning of antimicrobial and antiplatelet therapy, the girl showed clinical improvement. The changes in the mandible and skin were no longer so pronounced, and blood pressure gradually decreased. Skin therapy slowly helped to reduce the intensity of the rash. Normalization in inflammatory parameters and decrease of D-dimer value were recorded. Thrombocytosis and slightly elevated LDH and AST persisted. Control thyroid hormones were in the normal range.

A week after discharge, the girl was better, except for her blood pressure, which was still high, so we adjusted the therapy of amlodipine to 2 mg (0.2 mg/kg) twice daily.

Three weeks after the discharge, she stopped antibiotic therapy, and after 1 month she also stopped taking antihypertensive drug amlodipine. Follow-up echocardiography was normal. One month after discharge, the serology for SARS-CoV-2 was negative.

At the last follow-up, almost 1 year after the first admission, the girl was in a better mood, no rash was present, and there was no inflammation in her oral cavity, but six teeth in the lower jaw (73, 72, 71, 81, 82, and 83) were missing, and other teeth showed caries. Her laboratory results were normal, except for persistently positive IgG aCL (11 AUG) and IgG anti-ß2GPI antibodies (16 AUG).

## Discussion

Various oral cavity-related manifestations in patients with COVID-19 have been reported, including oral necrotic ulcers and aphthous-like ulcerations. These manifestations usually develop early in the disease course and affect the tongue, lips, palate, and oropharynx (8, 9). Aphthous changes on the tongue and buccally were also observed in our patient. The oral lesions in COVID-19 could be due to the interaction between SARS-CoV-2 and ACE-2 receptors, expressed on epithelial cells of the tongue and salivary glands, which might increase the permeability of the cell walls to foreign pathogens and viral replication in the cells lining the oral mucosa, leading to ulcers and necrosis (8).

Necrotizing periodontal disease was also described in association with COVID-19 (10). SARS-CoV-2 infection may predispose individuals to necrotizing periodontal disease through bacterial coinfection propagated by Prevotella intermedia (11), which was reported in a 35-year-old woman with necrotizing gingivitis due to Prevotella intermedia and COVID-19 (12). Moreover, there was a case report of a 38-year-old man who developed osteonecrosis of maxilla as a possible oral manifestation of COVID-19 (13). To our knowledge, our patient is the first reported case of NS with mandibular bone necrosis after SARS-CoV-2 infection in a child.

Endothelial dysfunction of multiple organs is a well-known feature of severe COVID-19 (14, 15). The SARS-CoV-2 virus binds to the angiotensin-converting enzyme-2 (ACE-2) receptor, which is also present on endothelial cells (16) and thus has a direct effect on them. It can also cause vasculitis and manifests as systemic inflammatory vascular disease (17). Several studies noted viral-like particles in endothelial cells (18, 19). Endothelial dysfunction may cause many of the symptoms by promoting inflammatory and microvascular thrombotic processes. Disruption of the endothelial barrier and endothelial cell function leads to vasoconstriction, increased vascular permeability, thrombosis, hyperinflammation, and hypertension (20).

In the present case, necrosis and endothelial damage with individual mural thrombi and in some cases the appearance of recanalization of thrombotic changes along small vessels were seen histopathologically. These changes could be linked to the hypercoagulable state and vascular damage in multisystem inflammatory response after SARS-CoV-2 infection. We found no SARS-CoV-2 viral particles in the biopsy samples. In contrast, Colmenero et al. (19) detected SARS-CoV-2 viral particles in endothelial cells in a skin biopsy of seven children with COVID-19 chilblains, and in one case viral particles were visible also on electron microscopy.

Different cutaneous manifestations of vasculitis have also been reported in patients with recent COVID-19 infection (21, 22). In our case, the girl had generalized erythematous maculopapular rash with additional petechiae in some localized areas. She had some clinical features of MIS-C and Kawasaki disease but did not fulfill criteria for either of these two diseases. High fever was present only for 1 day, and she had no conjunctivitis, no lymphadenopathy, and no cardiac involvement. Treatment with intravenous immunoglobulins was considered but was not given since the patient did not meet the criteria for MIS-C or Kawasaki disease. Moreover, application of intravenous immunoglobulin could increase blood viscosity and further reduce arterial and capillary blood flow.

Possible causes for hypertension in our patient were endothelial dysfunction due to swollen endothelial cells and luminal narrowing of the blood vessels or thyroid impairment. Renal artery stenosis was ruled out with a Doppler renal ultrasound. Hypertension persisted even after the normalization of thyroid hormones, so we hypothesize that arterial hypertension could be linked with endothelial dysfunction (21, 22).

Numerous autoimmune manifestations associated with COVID-19 are described in the literature including the presence of several different autoantibodies (23). Anticardiolipin and other antiphospholipid antibodies were frequently reported in patients with COVID-19 (24) and were associated with a more severe disease course (25). It is likely that the presence of aCL in our patient reflects endothelial damage due to SARS-CoV-2 infection and could additionally contribute to the procoagulant state. Our patient also had signs of thyroiditis, which has been reported before in association with SARS-CoV-2 infection (26).

The treatment of NS includes treatment of the acute phase with systemic antibiotics and sometimes with antifungal agents, which are important especially in immunosuppressed patients (27). The treatment of any possible preexisting condition is also important. To lower the possibility for relapses, long-term support should be obtained. In our patient, we obtained a satisfactory response with local and systemic treatment. The girl lost six deciduous teeth along with alveolar bone, and it is unknown at this time whether permanent teeth in this area will be affected. Mucosa healed without any scarring, and it appeared normal in structure and color.

One of the limitations of our case report is the impossibility to determine the causative role of COVID-19 in the disease. The cause of the girl's symptoms and signs could have been some other infection, but it was an unusual combination of clinical and laboratory features that was not previously reported and occurred at the peak of the SARS-CoV-2 epidemic. Moreover, the extensive infectious disease workup was negative except for positive SARS-CoV-2 serology.

Given the disease course, positive SARS-CoV-2 serology, and signs of endothelial cell swelling on the skin biopsy, it appears that NS in this patient was due to COVID-19. The complex pathogenic mechanisms of NS and bone necrosis linked with COVID-19 are still unclear but could be a consequence of inflammatory events with vasculopathy and damage of endothelial cells as a response to the SARS-CoV-2 infection.

## Data Availability Statement

The original contributions presented in the study are included in the article/supplementary material, further inquiries can be directed to the corresponding author/s.

## Ethics Statement

Written informed consent was obtained from the minor(s)' legal guardian/next of kin for the publication of any potentially identifiable images or data included in this article.

## Author Contributions

NE, TT, OT, MK and TA contributed in the patient care and treatment. NE and TA wrote the manuscript and reviewed the literature. DK contributed to interpreting and describing the imaging findings. MV contributed to describing histopathological evaluation. All authors contributed to diagnostic procedure, manuscript revision, read, and approved the submitted version.

## Funding

This work was partially supported by the Slovenian Research Agency Grant J3-3061 and University Medical Center Ljubljana Grant 20210069.

## Conflict of Interest

The authors declare that the research was conducted in the absence of any commercial or financial relationships that could be construed as a potential conflict of interest. The handling Editor declared a past co-authorship with one of the authors TA.

## Publisher's Note

All claims expressed in this article are solely those of the authors and do not necessarily represent those of their affiliated organizations, or those of the publisher, the editors and the reviewers. Any product that may be evaluated in this article, or claim that may be made by its manufacturer, is not guaranteed or endorsed by the publisher.
